# 73432 Assessment of multi-pollutant ambient air composition on type 2 diabetes mellitus using machine learning.

**DOI:** 10.1017/cts.2021.522

**Published:** 2021-03-30

**Authors:** Naomi Oiwa Riches, Ramkiran Gouripeddi, Julio C. Facelli

**Affiliations:** University of Utah

## Abstract

ABSTRACT IMPACT: We explored the use of machine learning to explore how multi-pollutant air quality is related to type 2 diabetes, which is more representative than the single pollutant models often employed to assess this relationship. OBJECTIVES/GOALS: Single pollutant air pollution models have correlated air pollution components with type 2 diabetes mellitus (DM). However, air pollution is a complex mixture, therefore, we explored the relationship between multi-pollutant air quality and DM incidence using machine learning. METHODS/STUDY POPULATION: Annual diabetes incidence from the CDC for each US county was downloaded for the years 2007-2016. Daily air pollution concentrations for PM2.5, PM10, CO, SO2, NO2, and O3 were downloaded from the US EPA for the years 2006-2015. K-means clustering, an unsupervised machine learning method, was employed to partition all air pollution components, for each day and county monitored, into the optimal number of clusters. Change in DM incidence was matched to air pollution clusters by county, lagged by one year. Additionally, NASA satellite-derived air pollution data will be compared to EPA data to inspect as a potential source for future clustering analysis of counties that do not have an EPA monitor. RESULTS/ANTICIPATED RESULTS: The largest increase of annual DM incidence was associated with the cluster having the highest average PM10, PM2.5, and CO, and the second greatest average NO2 concentrations. Inversely, the most significant decrease of annual DM incidence was associated with the cluster having the lowest PM10, PM2.5, and CO. While average PM10, PM2.5, SO2, NO2, and CO showed a rising tendency with elevating change of DM incidence, ozone did not show any such trend. It is anticipated that the NASA satellite-derived air pollution data will approximate the EPA air quality data and will be usable in assessing the air pollution-DM relationship for areas currently not monitored by the EPA. DISCUSSION/SIGNIFICANCE OF FINDINGS: Using an unsupervised k-means algorithm, we showed multiple ambient air components were related to increased incidence of T2DM even when average concentrations were below the National Ambient Air Quality Standards. This work could help guide policy making regarding air quality standards in the future.

